# Identification of the Annexin A2-interacting domain of pneumococcal PsaA

**DOI:** 10.1128/msphere.00232-26

**Published:** 2026-04-30

**Authors:** Prattay Dey, Yoonsung Hu, Faith Henson, Chaeyoung Kim, Nogi Park, Keun Seok Seo, Justin A. Thornton

**Affiliations:** 1 Department of Biological Sciences, Mississippi State University 5547, Mississippi State, Mississippi, USA; 2 Department of Comparative Biomedical Sciences, College of Veterinary Medicine, Mississippi State University, Mississippi State, Mississippi, USA; Vanderbilt University Medical Center, Nashville, Tennessee, USA

**Keywords:** pneumococcus, adhesion molecules, protein interactions

## Abstract

*Streptococcus pneumoniae* is a gram-positive bacterium, colonizer of the human nasopharynx capable of causing severe invasive disease. Colonization of the nasopharynx is a prerequisite for the development of invasive disease and depends upon surface-bound bacterial proteins interacting with host cell receptors. Pneumococcal surface adhesin A (PsaA) is a highly conserved lipoprotein involved in the attachment to host airway cells via the host receptor Annexin A2 (ANXA2). However, the specific structural domains of PsaA responsible for ANXA2 recognition and binding remain unknown. Here, we employed a structure-based peptide approach to map the ANXA2-binding domain of PsaA. Five recombinant PsaA peptides were designed and generated based on the crystal structure of PsaA. Far-western blot analysis using ANXA2-overexpressing HEK293T/17 cell lysates revealed that the interaction with ANXA2 was specific to the C-terminal subdomain of PsaA. Cell-binding assays and flow cytometry further confirmed the interaction between the C-terminal subdomain of PsaA and cell surface-expressed ANXA2. Furthermore, polyclonal antibodies against the C-terminal subdomain significantly inhibited the binding of full-length PsaA to ANXA2-overexpressing cells, whereas antibodies against other subdomains did not. Consistently, polyclonal antibodies against the C-terminus of PsaA reduced the binding of *S. pneumoniae* to A549 lung epithelial cells to a greater extent than other antibodies. Together, these findings establish that the C-terminus of PsaA is crucial for ANXA2 receptor recognition. Targeting this specific subdomain may be a promising strategy for developing next-generation protein-based pneumococcal vaccines that aim at blocking bacterial adherence.

**IMPORTANCE:**

*Streptococcus pneumoniae* is a leading cause of millions of deaths worldwide each year due to its ability to transition from an asymptomatic colonizer to an invasive pathogen. Current pneumococcal conjugate and polysaccharide vaccines protect against pneumococcal disease, but overall colonization rates have remained stable. Since pneumococcus is an opportunistic pathogen, decreasing overall colonization rates is essential for preventing progression to disease. The significance of our research lies in mapping functional epitopes within key pneumococcal adhesins that play a critical role in bacterial adherence. Defining these adhesion epitopes is essential for the rational design of next-generation protein-based vaccines capable of blocking colonization and ultimately reducing the global burden of invasive pneumococcal diseases.

## INTRODUCTION

*Streptococcus pneumoniae* is a gram-positive, facultative anaerobe and a major cause of global morbidity and mortality (1). It usually colonizes the human nasopharynx as a commensal but can act as an opportunistic pathogen by spreading in the host and causing invasive pneumococcal disease (IPD), such as bacteremia, meningitis, and pneumonia, as well as non-invasive infections like otitis media and sinusitis (2, 3). Nasopharyngeal colonization is the prerequisite first step in disease development, and colonized individuals are the main reservoir for community transmission (4, 5).

Current preventive strategies use polysaccharide-based vaccines, including the pneumococcal conjugate vaccine and the pneumococcal polysaccharide vaccine (6). Updated vaccine recommendation for adults in the United States includes PCV21 (Capvaxive) (7). Although these vaccines have effectively reduced disease from targeted serotypes, they have notable limitations for the inability to protect from community-acquired pneumonia (8). The adaptability of pneumococcus has led to serotype replacement, where non-vaccine serotypes fill the ecological niche left by vaccine-targeted strains (9, 10). Additionally, antibiotic-resistant clones among non-vaccine serotypes pose an increasing public health concern (11). These challenges have prompted the development of serotype-independent vaccines that target highly conserved surface proteins to provide broad-spectrum protection (12).

One candidate for inclusion in a protein-based vaccine is pneumococcal surface adhesin A (PsaA). PsaA is a 37-kDa lipoprotein and a component of an ABC transporter essential for manganese (Mn^2+^) acquisition (13). PsaA is expressed in most pneumococcal strains and is highly conserved and immunogenic in humans, making it a strong candidate for a universal vaccine (14–17). In addition to its role in oxidative stress resistance through manganese transport, PsaA has also been well documented as an adhesin, enabling bacterial attachment to the upper respiratory tract (13, 18–22). PsaA deletion mutants show significantly reduced adherence and decreased virulence in mammalian cells and murine models (23, 24).

To effectively target PsaA for vaccine development, it is essential to understand the molecular mechanisms underlying its interaction with host cells. Previous studies identified E-cadherin, a cell-cell adhesion protein, as a receptor for PsaA that promotes pneumococcal adherence to nasopharyngeal cells (25). More recently, our group identified human Annexin A2 (ANXA2) as a second, high-affinity receptor (26).

ANXA2 is a calcium-dependent, phospholipid-binding protein that is highly expressed on respiratory epithelial and endothelial cells. A number of pathogens are known to exploit ANXA2 as a host-surface receptor or as a receptor-like binding partner, though the extent of mechanistic understanding varies between pathogens. In *Rickettsiae* infection, outer membrane protein B (OmpB) serves as the microbial adhesin that binds to endothelial ANXA2. Su et al. demonstrated that Tyrosine 23 in the N-terminal region of ANXA2 is important for this interaction, but a minimal OmpB ANXA2-binding motif has not yet been identified (27, 28). During *Escherichia coli* infection, ANXA2 interacts with the bacterial Espl2, which promotes F-actin binding activity and drives membrane/cytoskeleton reorganization required for infection (29). In *Salmonella* infection, ANXA2 is recruited by the type III secretion system (T3SS) effector SopB, which is required for efficient bacterial invasion into cultured epithelial cells (30). In *Mycoplasma pneumoniae*, the CARDS toxin binds ANXA2 and functions as a receptor-mediated ligand, with binding and internalization activities localized to the carboxyl-terminal portion of the toxin, particularly the D2+D3 or D3 region. The precise ANXA2 contact site in this case remains unmapped (31, 32). Among viral pathogens, enterovirus 71 (EV71) utilizes capsid protein VP1 to bind ANXA2. This interaction is relatively well characterized as VP1 amino acids 40 to 100 have been identified as important for ANXA2 binding, and subsequent studies have linked this interaction to the fourth annexin repeat of ANXA2 (33). Additional pathogens have also been associated with ANXA2-dependent attachment or entry but with less complete definition at the domain level. For example, HPV16 L2 interacts with the ANXA2 heterotetramer (A2t), with the best-mapped viral region being L2 residues 108 to 120. This segment appears to bind primarily to S100A10/p11, the ANXA2 partner protein, rather than to ANXA2 alone, indicating an A2t-dependent rather than a strictly ANXA2-only interaction (34). Although multiple pathogens target ANXA2 as a host receptor, only a subset of these interactions has been mapped to a defined microbial binding region or a specific ANXA2 domain. Recently, we showed that blocking the PsaA-ANXA2 interaction significantly reduces pneumococcal adherence, indicating that this protein-protein interaction plays an important role in colonization (26). Although these receptor-ligand interactions have been identified, the specific structural domains within PsaA that mediate ANXA2 recognition remain undefined. Epitope mapping is essential for rational vaccine design because it enables the identification of functional domains that elicit neutralizing antibodies while avoiding non-protective or immunodominant epitopes (35). In this study, we employed a structure-based peptide mapping approach to define the PsaA domains involved in interaction with human ANXA2. By subdividing PsaA into the N-terminal domain, central helical linker, and C-terminal domain, we identified the C-terminal domain required for ANXA2 recognition. Our results demonstrate that the PsaA-ANXA2 interaction is domain-specific and is primarily mediated by the C-terminal domain of PsaA.

## RESULTS

### Structure-based design of PsaA peptides

**Fig 1:**
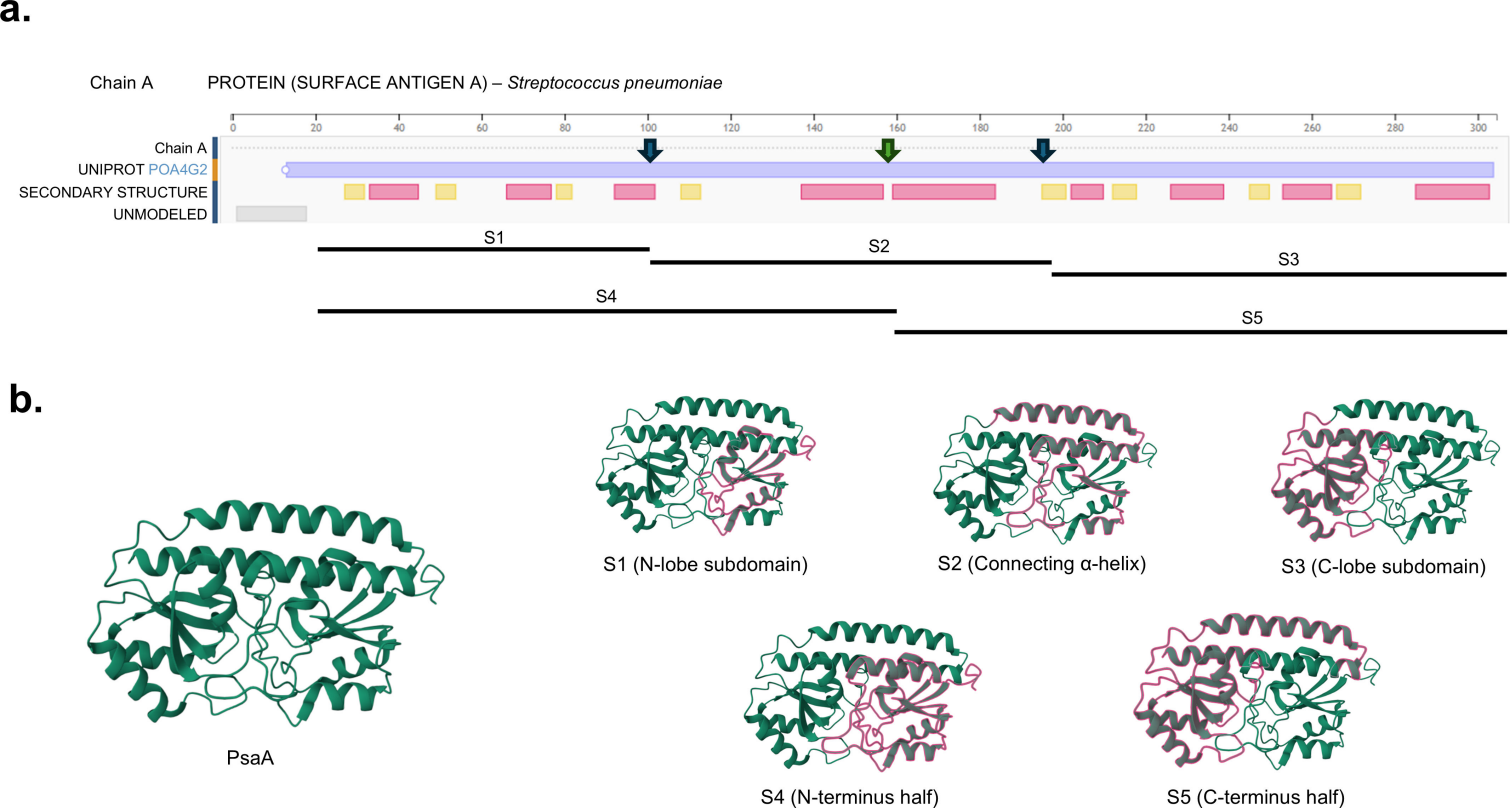
Crystal structure-based design of PsaA-derived peptides. (**a**) Linear representation of the pneumococcal surface adhesin A (PsaA; UniProt P0A4G2) showing the full-length amino acid sequence with annotated secondary structure elements. Arrows indicate designed peptides based on the secondary structure of the protein. (**b**) Three-dimensional ribbon model of full-length PsaA and the corresponding structure-based peptides. The full-length PsaA structure is shown on the left. Highlighted regions (pink) indicate peptides designed from distinct structural elements: S1, the N-terminal subdomain; S2, the central connecting α-helix; S3, the C-terminal subdomain; S4, the N-terminal half of PsaA; and S5, the C-terminal half. Green regions represent the remainder of the protein outside each peptide.

PsaA is a highly conserved lipoprotein consisting of three major domains: an N-terminal domain, a central α-helical linker, and a C-terminal domain. The N-terminal domain contributes to overall structural stability, while the central α-helical linker provides conformational flexibility and spatial separation between terminal domains. The C-terminal domain forms a distinct surface-exposed structure that is well-suited for interaction with host ligands (20). To determine which portions of PsaA interact with ANXA2, PsaA without the 19-amino acid signal peptide (289 amino acids in length) was subdivided into five peptides (Fig. 1). These peptides were designated as S1 (N-terminal subdomain, three α-helices and three β-sheets, amino acids 21–100; 240-bp amplicon), S2 (central α-helical connector, two α-helices, amino acids 101–190; 270-bp amplicon), S3 (C-terminal subdomain, four α-helices and four β-sheets, amino acids 191–309; 357-bp amplicon), S4 (N-terminal half, four α-helices and four β-sheets, amino acids 21–157; 411-bp amplicon), and S5 (C-terminal half, five α-helices and four β-sheets, amino acids 158–309; 456-bp amplicon).

All five gene regions encoding those peptides were individually cloned in the pET 100/D-TOPO vector, with forward primers possessing 5′-CACC overhangs for TOPO directional cloning. The recombinant peptides were expressed with a 6×-Histidine tag for purification, and protein expression was confirmed by western blot analysis. High purification of the peptides was demonstrated by SDS-PAGE (Fig. S1).

### Identification of PsaA-derived peptides binding to ANXA2 by far-western blot

**Fig 2:**
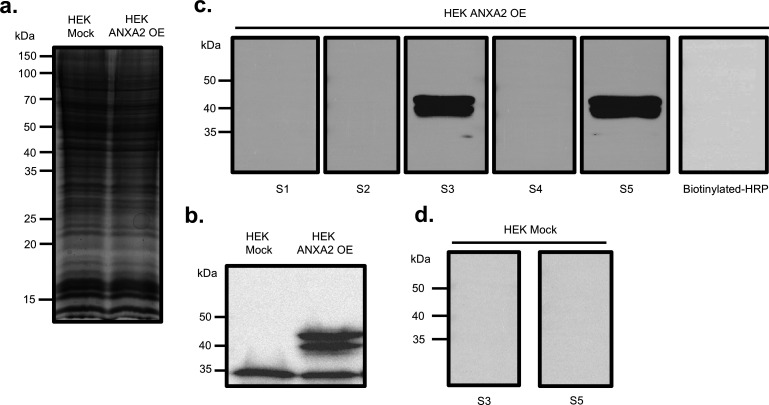
Far-western blot analysis of PsaA-derived peptide interaction with ANXA2. (**a**) Coomassie-stained SDS-PAGE gel of HEK 293 T/17 Mock and ANXA2-overexpressing (ANXA2 OE) cell lysates. (**b**) Western blot of ANXA2 OE and HEK 293 T/17 Mock cell lysates probed with anti-ANXA2 antibody. (**c**) Far-western blot of ANXA2 OE cell lysates probed with biotinylated PsaA-derived peptides (S1–S5) and ANXA2 OE cell lysates probed with biotinylated-HRP (negative control). (**d**) Far-western blot repeated with HEK 293 T/17 Mock cell lysates probed with biotinylated S3 and S5 peptides. All biotinylated proteins were detected with streptavidin-HRP.

To assess interactions between PsaA-derived peptides with ANXA2, whole-cell lysate proteins from mock-transfected HEK 293 T/17 cells (HEK 293 T/17 Mock) and HEK 293 T/17 ANXA2-overexpressing (ANXA2 OE) cells were separated on a 12.5% gel by SDS-PAGE. Proteins were visualized by Coomassie blue staining to confirm efficient protein extraction and separation (Fig. 2a). To confirm the ANXA2 expression, proteins were transferred to a polyvinylidene fluoride (PVDF) membrane and probed with an anti-ANXA2 primary antibody. ANXA2 expression was detected using a horseradish peroxidase (HRP)-conjugated secondary antibody. Strong ANXA2 expression was observed as doublet bands approximately 40 kDa in lysates from ANXA2 OE cells, whereas no detectable ANXA2 signal was observed in HEK 293 T/17 Mock cells (Fig. 2b). The doublet band of ANXA2 can be routinely seen with the primary antibody used for detection of ANXA2 (36). Notably, a band approximately at 35 kDa was detected in both ANXA2 OE and HEK 293 T/17 Mock cell lysates and was therefore considered nonspecific.

For far-western blot analysis, proteins were transferred to PVDF membranes and probed with each of five biotinylated PsaA-derived peptides (S1–S5). Bound peptides were detected by HRP-conjugated streptavidin. Among these peptides, S3 and S5 exhibited distinct doublet bands at approximately 40 kDa, corresponding to ANXA2, whereas the other three peptides (S1, S2, and S4) showed no detectable interaction. Consistently, neither HRP-conjugated streptavidin control (Fig. 2c, far-right panel) nor lysates from HEK 293 T/17 Mock cell (Fig. 2d) exhibited detectable binding in the far-western blot analysis.

### ANXA2-dependent binding of PsaA and PsaA-derived peptides by cell binding assay

To determine the extent to which full-length PsaA and PsaA-derived peptides can bind to cell surface-expressed ANXA2 and whether anti-sera against S1 or S3 can inhibit PsaA interactions with ANXA2, ANXA2 OE and HEK 293 T/17 Mock cells were incubated with full-length PsaA, PsaA-derived peptides (S1 or S3), and full-length PsaA preincubated with anti-S1 or S3 polyclonal antibody. After incubation, cells were lysed, and bound PsaA or peptides were detected by western blot using anti-PsaA polyclonal antibody.

**Fig 3:**
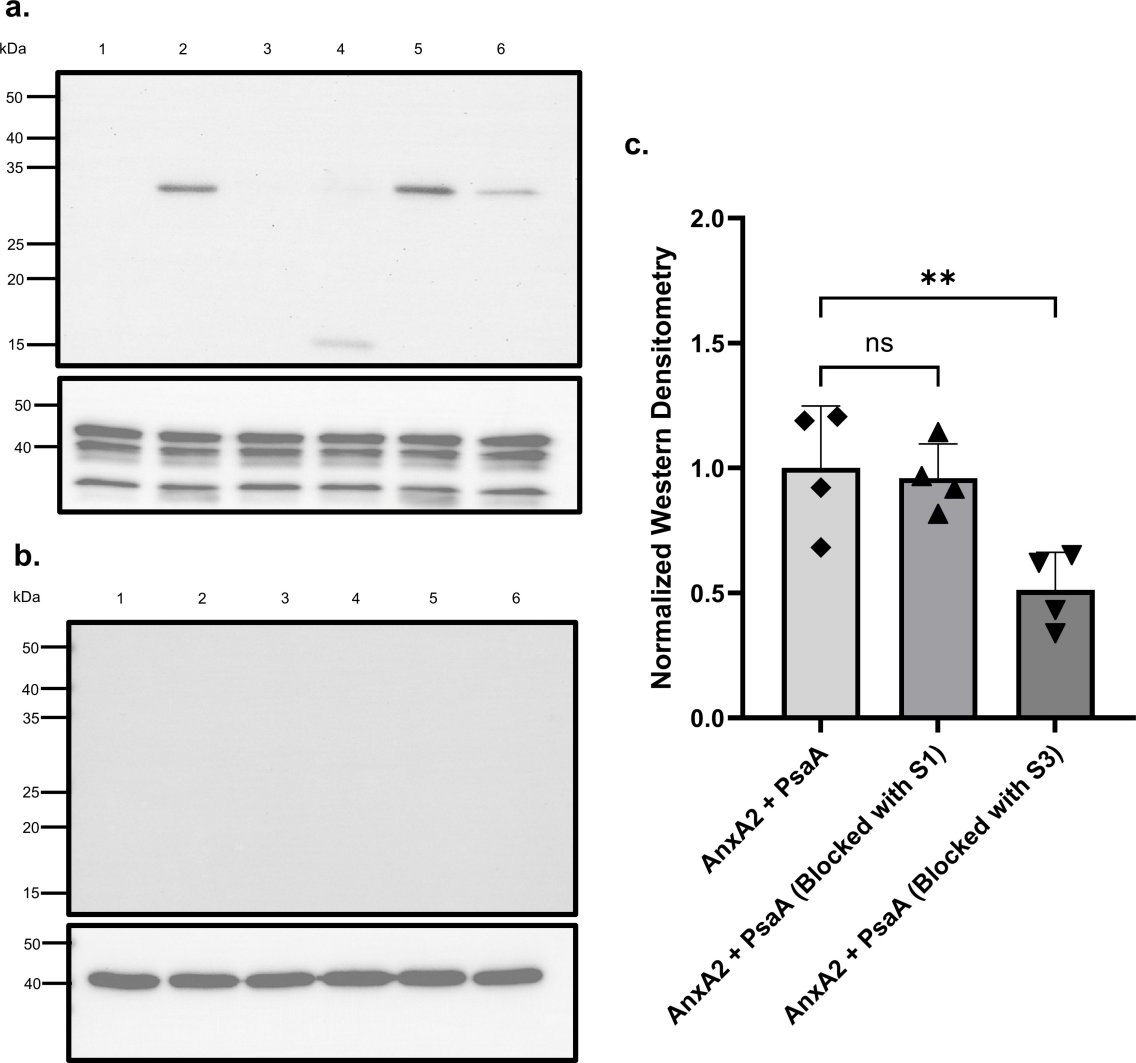
Cell binding assay demonstrating ANXA2-dependent interaction of PsaA and PsaA-derived peptides. (**a**) HEK 293 T/17 ANXA2 (ANXA2 OE) cells were incubated with purified PsaA (Lane 2), PsaA-derived peptides (Lane 3, S1 and Lane 4, S3), and PsaA preincubated with anti-peptide sera (Lane 5, anti-S1 or Lane 6, anti-S3). Bound proteins were detected by western blot using anti-PsaA serum. Lane 1 (cells only) served as a negative control. The lower panel represents ANXA2 protein levels (loading control). (**b**) Duplicate experiment using HEK 293 T/17 Mock cells lacking ANXA2 expression. No detectable PsaA or peptide binding was observed under any condition. Lower panel showing β-actin (loading control). (**c**) Quantification of the binding intensity of PsaA to ANXA2 by western blot densitometry.

Strong binding was observed in ANXA2 OE cells (Fig. 3a) incubated with full-length PsaA (Fig. 3a, lane 2) and the PsaA S3 peptide (Fig. 3a, lane 4), whereas no detectable signal was observed in ANXA2 OE cells incubated with the PsaA S1 peptide (Fig. 3a, lane 3). These results indicated that PsaA binding to cell surface-expressed ANXA2 is specific to the C-terminal domain. Preincubation of full-length PsaA with anti-S3 sera (Fig. 3a, lane 6) resulted in a partial reduction in band intensity, whereas preincubation with anti-S1 sera (Fig. 3a, lane 5) resulted in no or minimal change. In contrast, no binding was observed in HEK 293 T/17 Mock cells (Fig. 3b). To ensure differences in binding were not due to variations in ANXA2 across samples, we also probed for ANXA2 (lower panel in Fig. 3a). This demonstrated equal ANXA2 across all samples and also served as a loading control. β-actin served as a loading control for Mock cells since they express negligible ANXA2 (lower panel in Fig. 3B). Western blot densitometry normalized to internal loading control showed that blocking PsaA with anti-S1 sera did not reduce the binding to ANXA2 significantly (*P* = 0.9363). On the other hand, blocking PsaA with anti-S3 sera significantly (*P* = 0.0087) reduced the binding of PsaA to ANXA2 (Fig. 3c). Collectively, these findings demonstrate that PsaA binds to cell surface-expressed ANXA2 via its C-terminal domain and that antibodies directed against the S3 region, but not the S1 region, can neutralize the PsaA-ANXA2 interaction.

### ANXA2-dependent binding of PsaA and PsaA-derived peptides measured by flow cytometry

**Fig 4:**
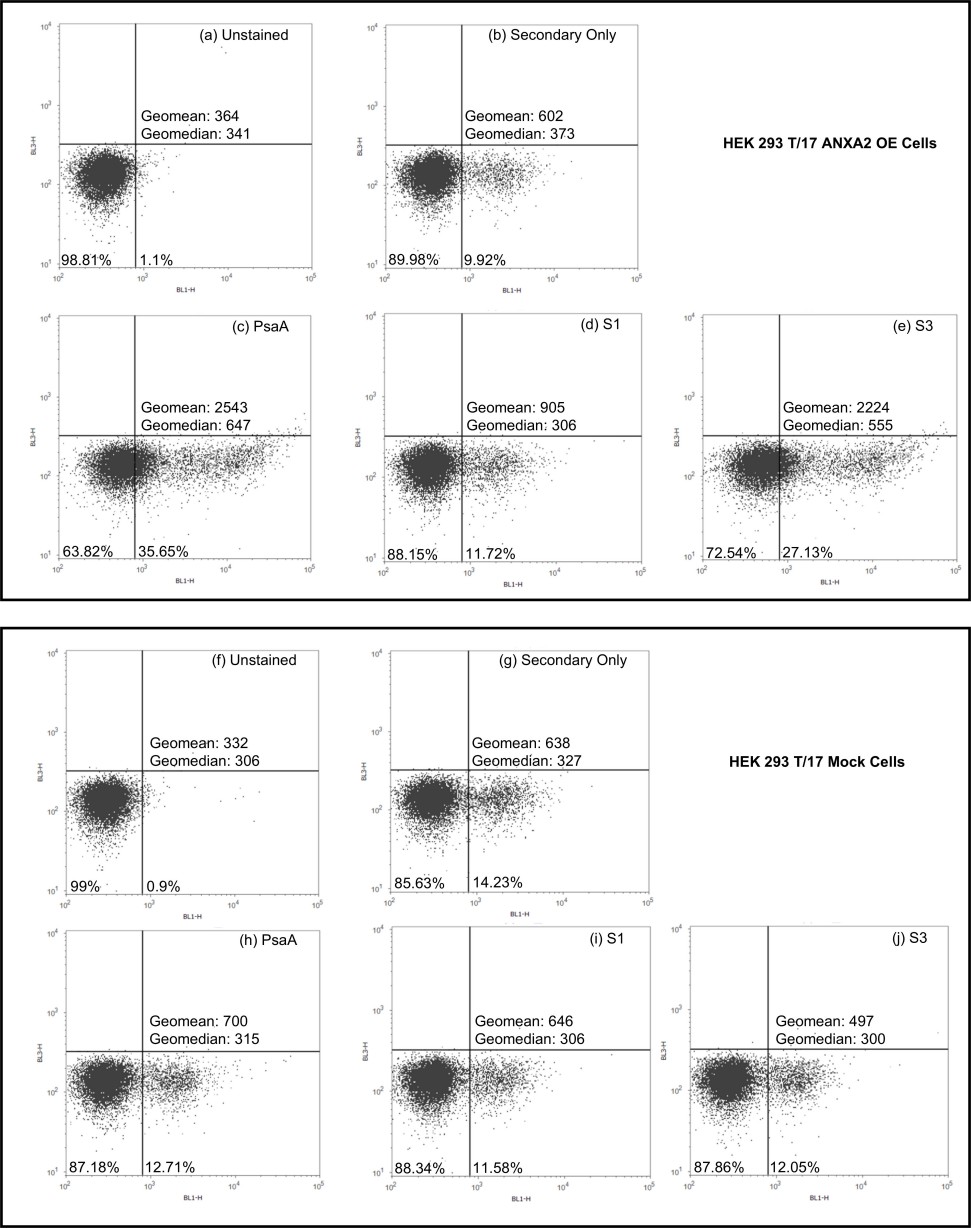
ANXA2-dependent binding of PsaA and PsaA-derived peptides assessed by flow cytometry. Flow cytometry density plots with the binding of full-length PsaA and PsaA-derived peptides to HEK 293 T/17 ANXA2 OE cells (top panel) and HEK 293 T/17 Mock cells (bottom panel). Cells were analyzed under the following conditions: (**a,f**) unstained cells, (**b,g**) secondary antibody-only controls, (**c,h**) PsaA, (**d,i**) S1 peptide, and (**e,j**) S3 peptide. Fluorescence intensity was measured in the BL1-H channel. Gates were set based on unstained and secondary-only controls, and the percentage of BL1-H-positive cells is indicated in each plot. Geometric mean fluorescence intensity (geomean) and geometric median fluorescence intensity (geomedian) values are shown for each condition.

To further validate the interaction between PsaA-derived peptides and surface ANXA2 under native cellular conditions, flow cytometry was performed using ANXA2 OE and HEK 293 T/17 Mock cells. Following incubation with full-length PsaA or PsaA-derived peptides, cells were stained with anti-PsaA sera followed by Alexa488-tagged goat-anti-mouse IgG. Unstained and secondary antibody-only samples were used to establish baseline fluorescence and gating parameters (Fig. 4a, b, f and g).

Consistent with the previous findings, incubation of ANXA2 OE cells with full-length PsaA resulted in increased fluorescence intensity, with a clear rightward shift in the BL1-H channel and 35.65% of cells falling within the positive gate (Fig. 4c). Similarly, incubation with the S3 peptide showed a distinct fluorescence shift, with 27.13% BL1-H-positive and an elevated geometric mean fluorescence intensity, indicating a strong interaction between the S3 region of PsaA and ANXA2 (Fig. 4e). In contrast, incubation with the S1 peptide resulted in only a minor shift in fluorescence, with a small proportion of cells in the positive gate (11.72%), similar to background levels observed in control samples (Fig. 4d).

To evaluate whether the observed binding was dependent on ANXA2 expression, identical binding assays were performed using HEK 293 T/17 Mock cells. In these cells, neither the full-length PsaA nor the S1 or S3 peptides produced a measurable increase in fluorescence relative to secondary-only controls (Fig. 4h through j). The absence of a fluorescence shift across all conditions indicates that binding of PsaA and the S3 peptide is dependent on ANXA2 expression and does not occur nonspecifically on the cell surface.

### Effect of PsaA-peptide-derived serum on pneumococcal adherence

**Fig 5:**
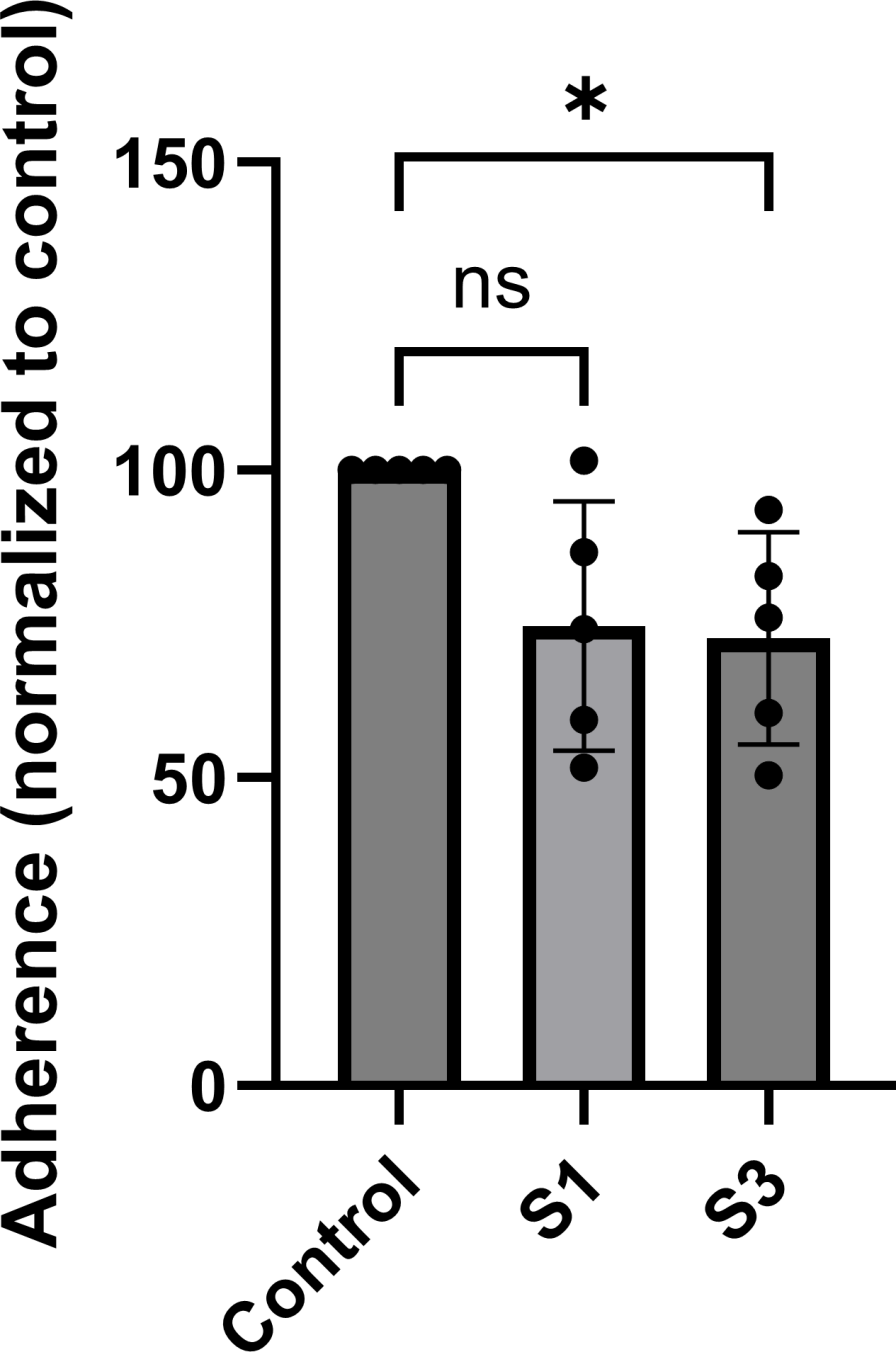
Effect of PsaA-peptide-derived serum on pneumococcal adherence in adhesion assay. Quantification of bacterial adherence to A549 cells. Bacterial adherence to A549 cells following pre-incubation with either anti-S1 or anti-S3 immune sera and normalized to control mouse sera.

To determine the effect of PsaA binding to ANXA2 on pneumococcal adherence, A549 lung epithelial cells were incubated with the unencapsulated *S. pneumoniae* JS1 strain (37) preincubated with normal mouse serum (control), anti-S1, or anti-S3 sera. Preincubation with anti-S3 sera significantly decreased bacterial adherence to A549 cells compared to preincubation with non-immune mouse sera (*P* = 0.0389). Preincubation with anti-S1 sera also resulted in a reduced adherence, but this was not significant (*P* = 0.0559) (Fig. 5). These results indicate that antibodies targeting the C-terminal (S3) region of PsaA can significantly inhibit pneumococcal adherence to A549 cells compared to antibodies targeting the N-terminal (S1) region.

## DISCUSSION

The ability of *S. pneumoniae* to cause disease depends mainly on its capacity to adhere to and invade host mucosal surfaces (5). A range of surface-exposed proteins facilitate this process. PsaA is recognized as a critical virulence factor required for effective colonization. However, the molecular details underlying its interaction with host cell receptors have not been fully resolved. In the present study, we analyzed the structural domains of PsaA to clarify its interaction with the host receptor ANXA2. Our results indicate that PsaA binds to ANXA2 in a domain-specific manner, with the interaction localized mainly to the C-terminal subdomain, corresponding to the S3 peptide. Additionally, we show that antibodies directed against this subdomain block PsaA adherence to host cells. These findings suggest that the C-terminal subdomain of PsaA may serve as a promising target for subunit vaccine strategies.

PsaA is a lipoprotein that belongs to the Cluster 9 family of ABC transporter substrate-binding proteins, characterized by a bi-lobe structure linked by a rigid α-helical backbone. Anderton et al. showed that PsaA acts as an adhesin and binds to E-cadherin (25); however, the specific PsaA epitope responsible for E-cadherin recognition has not yet been identified. Moreover, the biological relevance of PsaA-E-cadherin interaction is uncertain given that E-cadherin is predominantly localized to intercellular junctions (38), rather than the apical epithelial surface. In contrast, ANXA2 is abundantly expressed on respiratory epithelial and endothelial cell surfaces and is known to be exploited by multiple invasive bacterial pathogens, making it a biologically plausible receptor for pneumococcal colonization.

Previous studies have relied on full-length PsaA protein, making it difficult to determine the specific roles of individual structural domains. To address this, we designed overlapping peptides based on the PsaA crystal structure to more precisely define the regions involved in the adhesin-receptor interaction. Far-western blot analysis demonstrated that only peptides containing the C-terminal subdomain region (S3 and S5) were able to interact with ANXA2. In contrast, the N-terminal subdomain (S1) and the central helical linker (S2) and the overlapping region of S1 and S2 (S4) did not bind. These findings indicate that the unique arrangement of β-sheets and α-helices in the C-terminal subdomain forms a conformational epitope that is necessary for ANXA2 recognition.

The identification of ANXA2 as the specific receptor for the PsaA C-terminal subdomain represents a significant finding in understanding pneumococcal pathogenesis. ANXA2 is a phospholipid-binding protein that is frequently upregulated on the surface of respiratory epithelial and endothelial cells, where it serves as a receptor for plasminogen and tissue plasminogen activator (39). He et al. showed that ANXA2 present on the luminal surface of the vascular endothelial cells acts as a host receptor and binds to bacterial rickettsial outer membrane protein B (*ompB*). They also demonstrated that when using ANXA2-null mice in *in vivo* studies, the lack of ANXA2 significantly reduced the bacterial adhesion. *Rickettsia* facilitates bacterial entry into endothelial cells utilizing the ANXA2-*ompB* interaction and plays a crucial role in early stages of bloodstream infection (27). Our findings suggest that *S. pneumoniae* utilizes the PsaA C-terminal subdomain to interact with ANXA2, which may contribute to the bacterium’s ability to adhere to the nasopharyngeal epithelium and possibly invade sterile body sites. Notably, the specificity of this interaction was demonstrated by the absence of binding in HEK 293 T/17 Mock cells, showing that PsaA adherence in this study is entirely dependent on ANXA2.

PsaA belongs to a highly conserved group of surface proteins known as the LraI family, many of which have been shown to similarly act as adhesins (40). These include FimA and SsaB of *Streptococcus parasanguinis* and ScsA of *Streptococcus gordonii*. The C-terminal region of PsaA is highly conserved across LraI family homologs and lacks canonical adhesin motifs, suggesting that host interaction is mediated by conformational surface features. Several conserved, surface-exposed charged patches (e.g., EEE-, DDR-, and KEGDS-containing regions) are present in this domain and may contribute to host binding. This is consistent with the binding properties of ANXA2, which interacts with ligands through Ca²^+^-dependent electrostatic interactions with anionic surfaces and hydrophobic contacts involving amphipathic structural elements rather than defined sequence motifs (41, 42). These observations support a model in which PsaA engages host proteins via conserved, structurally defined surface patches rather than linear adhesin motifs. However, targeted mutagenesis of conserved regions would be required to validate the contribution of such regions to ANXA2 binding.

A key observation from our study is the functional blocking activity exhibited by anti-S3 antibodies. In cell-binding assays, pre-incubation of full-length PsaA with anti-S3 serum led to a significant reduction in PsaA binding to ANXA2 OE cells, whereas anti-S1 serum did not produce an inhibitory effect (Fig. 3). These results indicate that the C-terminal subdomain serves as the primary ANXA2-interacting domain. The partial inhibition observed in our experiments is likely due to the structural context of the S3 region within the full-length PsaA protein. It has been demonstrated that the overall protein conformation undergoes subtle rearrangements upon metal binding. Due to this structural rigidity and the limited conformational flexibility, the S3-containing domain may not be consistently surface-exposed or readily accessible to antibody binding in the native protein. Also, metal-free PsaA is conformationally dynamic, with the C-terminal lobe showing flexibility, supporting the idea that manganese availability could influence the exposure of functional interaction surfaces. Consequently, antibodies generated against the isolated S3 peptide may have limited access to the corresponding epitope in the full-length protein, resulting in only partial, rather than complete inhibition (19, 43).

Protein interactions in solution and blotting of denatured proteins can occasionally result in artifact findings that are not indicative of actual protein interactions in living cells. Therefore, we performed flow cytometry to quantitate actual protein bound to surface ANXA2 on live cells as well as adhesion assays to evaluate the ability of domain-specific antibody to block surface ANXA2-dependent bacterial attachment. Flow cytometry clearly demonstrated that full-length PsaA and S3 peptide bind to the surface of ANXA2 OE cells and not HEK 293 T/17 Mock cells, indicating the interaction was dependent upon ANXA2. Similarly, there was a significant reduction in the adherence of *S. pneumoniae* to A549 cells when the bacteria were pretreated with anti-S3 sera as compared to non-immune sera or anti-S1 sera. Importantly, the results of both experiments are not due to greater titers of antibodies against S3 as compared to S1. This is evident by the fact that anti-full length PsaA antisera reacted to a greater degree against S1 versus S3 (Fig. S2). Additionally, when equal concentrations of S1 and S3 were blotted and probed with their respective antisera, we saw similar band intensities (Fig. S3). This indicates that antibodies against the S3 domain are qualitatively better at blocking the PsaA-ANXA2 interaction.

In our study, we found that all four peptides were immunogenic, except peptide S2, which could not be detected with the anti-S2 sera in a western blot (Fig. S2). Anti-S1 and anti-S3 serum titer was normalized by loading an equal concentration of S1 and S3 peptides on a SDS-PAGE gel and performing a western blot by probing with respective sera at a 1:2,500 dilution. However, the S2 peptide could not be detected with anti-S2 sera (Fig. S3). Our data indicate that only the C-terminal subdomain contributes to ANXA2-mediated adhesion. This distinction between immunogenicity and functional relevance is a well-recognized challenge in selecting vaccine antigens. The N-terminal subdomain may serve alternative roles, including stabilizing the solute-binding cleft required for manganese uptake.

It is important to recognize that the use of recombinant peptides may not fully replicate the tertiary structure of the native protein as it is anchored within the bacterial membrane. Nevertheless, the consistency observed between our interaction study results and our cell-binding assays utilizing live cells provides strong support for the S3 domain as the principal binding site. Future investigations will aim to identify the specific amino acid residues within the C-terminal subdomain that mediate this interaction and to assess the protective capacity of the S3 peptide in *in vivo* models of colonization. Romera-Steiner et al. showed that their designed P4 epitope, containing homologous amino acid residues (251–278) of PsaA, significantly affected adherence to nasopharyngeal epithelial cells. Also, when human serum was added, it led to the inhibition of the adherence of the peptide to Detroit 562 cells (44). Wang et al. demonstrated that when mice were immunized with engineered recombinant live-attenuated *Salmonella* vaccine expressing either full-length PsaA (aa 1–309) or different truncated versions of PsaA (aa 1–210), only full-length PsaA produced high serum IgG titers and strong mucosal IgA response. When immunized mice were further challenged with pneumococci, only the full-length protein containing the C-terminal region (aa 211–309) significantly reduced nasal colonization in both C57BL/6 and BALB/c mice. Truncated PsaA covering aa 1–210 did not reduce colonization and could not generate high anti-PsaA serum IgG and mucosal IgA titers, indicating the presence of minimal protective efficacy (45). All of these findings strengthen our data, in which we found the C-terminal subdomain (aa 191–309) of PsaA is responsible for interaction with host ANXA2, and targeting this region during subunit vaccine design might be crucial to reduce nasopharyngeal colonization.

In summary, this study presents the first comprehensive mapping of the PsaA-ANXA2 interaction, establishing the C-terminal subdomain as the key determinant for host receptor binding. These results advance our understanding of pneumococcal pathogenesis and indicate that the C-terminal subdomain is a prime target for PsaA-based vaccines to disrupt bacterial adherence and limit disease progression.

## MATERIALS AND METHODS

### Design of pneumococcal surface antigen A (PsaA) peptides

The pneumococcal surface adhesin A (PsaA) protein of *Streptococcus pneumoniae* (GenBank accession no. AAK75729.1) served as the template for peptide design in this study. The complete 309 amino acid sequence of PsaA was obtained from the NCBI database and analyzed to identify regions with distinct structural and functional properties. By analyzing the three-dimensional structure of PsaA (UniProt ID: P0A4G2), the N-terminal subdomain, C-terminal subdomain, and the core α-helical region linking the two lobes could be identified. Fig. 1 illustrates the design of five peptides (S1–S5) represented by their domain organization.

Peptide S1 was designed to correspond to the N-terminal subdomain, while S2 represents the central α-helical connecting region, and S3 corresponds to the C-terminal subdomain. In order to achieve broader structural coverage, S4 and S5 were constructed to represent the N-terminal and C-terminal halves of the protein, respectively.

### Cloning of PsaA peptides

The PsaA peptide genes were amplified from TIGR4 chromosomal DNA with peptide gene-specific primers in Table S1 using Q5 high-fidelity DNA polymerase (New England Biolabs, Cat# M0492S) following the manufacturer’s protocol. The genes were cloned using the Champion pET100 Directional TOPO Expression Kit (Invitrogen, Cat# K10001). PCR products were purified using the DNA Clean & Concentrator-25 kit (Zymo Research, Cat# D4034). Each peptide gene was ligated using a 2:1 ratio of clean PCR product to pET 100/D-TOPO vector and provided salt solution based on the manufacturer’s protocol. The ligation mixture was transformed into One Shot TOP10 Chemically Competent *E. coli* (Invitrogen, Cat# C404003) cells and incubated with shaking for 1 h in Super Optimal broth with Catabolite repression (S.O.C) media. Transformants were spread on Luria-Bertani (LB) agar plates containing 200 μg/mL of ampicillin (Amp) and incubated overnight at 37°C. Several colonies were picked for colony PCR, and the positive clones were confirmed using T7 promoter forward (5´-TAATACGACTCACTATAGGG −3´) and reverse (5´-GCTAGTTATTGCTCAGCGG-3´) primers, using PCRBIO Taq DNA polymerase (PCRBIO, Cat# PB10.23-02). Positive *E. coli* transformants were grown with shaking in LB media containing 200 µg/mL ampicillin at 37°C overnight. The positive pET100/D-TOPO vectors containing the peptide genes were individually extracted from *E. coli* using the Zyppy plasmid miniprep kit (Zymo Research, Cat# D4036). The positive vectors were further transformed into One Shot BL21 Star(DE3) chemically competent *E. coli* (Invitrogen, Cat# C601003) cells and incubated with shaking for 1 h in S.O.C. media. After that, 100 µL of transformed bacterial culture was spread on an LB agar plate (ampicillin: 200 μg/mL) and incubated overnight at 37°C. A single colony was picked for colony PCR, and the positive clone was confirmed using T7 promoter forward and reverse primers. Colony PCR was performed using PCRBIO Taq DNA polymerase (PCRBIO, Cat# PB10.23-02). Positive *E. coli* transformant was grown with shaking in LB media containing 200 µg/mL of ampicillin at 37°C overnight and frozen at −80°C for later use.

### Expression and purification of PsaA peptides

To express each peptide, a single BL21 *E. coli* colony containing the appropriate recombinant plasmid was inoculated into Luria-Bertani (LB) medium supplemented with 200 µg/mL ampicillin and incubated overnight at 37°C with shaking at 200 rpm. The overnight culture was subsequently diluted 1:100 into 100 mL of fresh LB medium containing ampicillin (200 µg/mL) and incubated at 37°C with shaking at 250 rpm until the optical density at 600 nm (OD_600_) reached approximately 0.6. At this point, protein expression was induced by the addition of 0.5 mM isopropyl β-D-thiogalactopyranoside (IPTG) for 3 h. Bacterial cells were collected from the 100 mL culture by centrifugation at 13,000 rpm for 10 min. The resulting cell pellet was resuspended and lysed in *E. coli* lysis buffer (20 mM Tris-HCl, 100 mM imidazole, 500 mM NaCl, 5% glycerol, at pH 7.95). Peptides were then purified from the lysate using HOOK 6X His Protein Spin Purification (Bacteria) with cobalt resin (G-Biosciences, Cat# 786-629) according to the manufacturer’s instructions.

### Biotin labeling of PsaA peptides

The purified peptides were biotinylated using EZ-Link Sulfo-NHS-LC-Biotin (ThermoFisher Scientific, Cat#. A39257) according to the manufacturer’s instructions. The excess nonreacted biotin reagent was removed by dialysis overnight at 4°C in phosphate-buffered saline (PBS) using Tube-O-DIALYZER, Medi, 8K MWCO (G-Biosciences, Cat# 786-617). Successful biotinylation was confirmed by western blot using Pierce High Sensitivity Streptavidin-HRP (ThermoFisher Scientific, Cat# 21130).

### Lentiviral expression of ANXA2 in the HEK 293 T/17 cell line

HEK293T/17 cells were acquired from the American Type Culture Collection (ATCC) and maintained in Dulbecco’s modified Eagle medium (DMEM; ATCC, Cat# 30-2002). The culture medium was supplemented with 10% fetal bovine serum (FBS) (R&D systems, Cat# S11550) 2 mM Gibco L-glutamine (ThermoFisher Scientific, Cat# 25030149), and HyClone Penicillin-Streptomycin (Cytiva, Cat# SV30010) to support optimal cell growth. The lentiviral expression plasmid pLenti-C-Myc-DDK, which encodes human annexin A2 (NM_001002858, pLenti-ANXA2), was sourced from Origene (RC215009). Lentiviral particles were generated by transfecting HEK293T/17 cells with pLenti-ANXA2 in combination with the packaging plasmid psPAX2 (Addgene) and the envelope plasmid pMD2.G (Addgene), utilizing the TransIT_293 transfection reagent (Mirus). This combination of plasmids ensured efficient packaging and production of the lentiviral vectors. Following transfection, the culture supernatant containing lentiviral particles was collected at 24, 48, 72, and 96 h post-transfection. The harvested supernatant was subsequently filtered through a 0.45-μm filter to remove cellular debris. Fresh HEK293T/17 cells were then transduced with the filtered lentiviral particles for 12 h. After transduction, cells were cultured overnight in 48-well plates. The following day, cells were transferred to 12-well plates and cultured overnight to allow for further expansion. On the third day, selection for ANXA2-expressing cells was performed by culturing in DMEM containing 5 µg/mL puromycin. As a negative control, HEK293T/17 Mock cells were generated using the empty transfer plasmid, following the same protocol as described above. To confirm successful expression of ANXA2, both Mock and overexpressing cell lines were lysed using RIPA buffer (ThermoFisher Scientific, Cat# 89901) according to the manufacturer’s instructions. The resulting lysates were analyzed by western blotting using an ANXA2 antibody (Santa Cruz, Cat# SC-28385), followed by detection with a goat anti-mouse IgG HRP-conjugated secondary antibody (Bio-Rad, Cat# 172-1011).

### Far-western blot

HEK 293 T/17 cells overexpressing ANXA2 were lysed, and total protein lysates were mixed with 4× Laemmli sample buffer (Bio-Rad, Cat# 1610747) and boiled at 95°C for 5 min to denature the proteins. Samples were separated by 12.5% sodium dodecyl sulfate-polyacrylamide gel electrophoresis (SDS-PAGE) at a constant voltage of 180 V. The resolved proteins were transferred to an Immobilon-P polyvinylidene fluoride (PVDF) membrane (Millipore Sigma, Cat# IPVH00010) using a semi-dry transfer system (Trans-Blot SD Cell, Bio-Rad, Cat# 1703940) with Rapid Transfer Buffer (VWR, Cat# 97064-314). Following transfer, membranes were rinsed with PBST buffer (137 mM NaCl, 2.7 mM KCl, 10 mM Na₂HPO₄, 1.8 mM KH₂PO₄, and 0.1% [wt/vol] Tween-20) and stained with Ponceau S (Thermo Fisher Scientific, Cat# 161470250) to confirm efficient protein transfer. Membranes were then blocked with PBST containing 5% non-fat, biotin-free dry milk (Fisher Scientific, Cat# NC9121673) for 1 h at room temperature. The blocked membrane was divided into five identical sections, each containing equivalent cell lysate lanes. Individual membrane pieces were incubated overnight at 4°C with gentle shaking in 5% milk prepared in PBST containing biotinylated peptides (50 µg each). The following day, membranes were washed with PBST and incubated with streptavidin-HRP (1:25,000 dilution) prepared in 5% milk for 1 h at room temperature. Bound peptides were detected using SuperSignal West Pico PLUS Chemiluminescent Substrate (Thermo Fisher Scientific, Cat# 34577), and signals were visualized using a ChemiDoc Imaging System (Bio-Rad, Cat# 12003153).

### Generation of anti-PsaA antibodies

Recombinant PsaA was purified using a *Staphylococcus aureus* expression system, as previously described (26). Purified PsaA peptides (S1–S3) and full-length PsaA were quantified using a Pierce bicinchoninic acid (BCA) assay (ThermoFisher Scientific, Cat#23227). For immunization, a final dose of 10 µg of protein and peptides was prepared for injection per mouse. Before administration, protein or peptides were emulsified with an equal volume of Imject alum adjuvant (ThermoFisher Scientific, Cat#77161). The emulsion of adjuvant and antigen (300 µL) was injected into C57BL/6 mice intraperitoneally. After initial immunization, mice were boosted twice at 2-week intervals between each dose. Antisera were collected from mice via the retro-orbital route 1 week after the final immunization and stored at −80°C for immunogenic assays.

### Cell binding assay

ANXA2 OE cells were cultured in T-150 flasks (TPP, Cat# 90150) to approximately 90% confluency. Cells were washed once with ice-cold PBS and detached using Gibco Cell Dissociation Buffer, enzyme-free, PBS (ThermoFisher Scientific, Cat# 13151014) at 37°C for 20 min. Detached cell layers were collected, washed twice with ice-cold PBS, and resuspended in PBS. Cell density was determined using a hemocytometer. For binding assays, 1 × 10⁶ cells were aliquoted into six 1.5 mL microcentrifuge tubes. Experimental conditions included the following: (i) HEK 293 T/17 ANXA2 OE cells only, (ii) HEK 293 T/17 ANXA2 OE cells incubated with recombinant PsaA, (iii) HEK 293 T/17 ANXA2 OE cells incubated with the S1 peptide, (iv) HEK 293 T/17 ANXA2 OE cells incubated with the S3 peptide, (v) HEK 293 T/17 ANXA2 OE cells incubated with PsaA preincubated with anti-S1 serum, and (vi) HEK 293 T/17 ANXA2 OE cells incubated with PsaA preincubated with anti-S3 serum. For assays involving serum preincubation, 10 µg of PsaA in PBS was mixed with anti-S1 or anti-S3 serum (1:50 dilution) and rotated at room temperature for 30 min to block the binding sites before adding to the cells. Cell suspensions were centrifuged at 500 × *g* for 5 min, and supernatants were discarded. The respective ligands or control solutions were added to the cells. For all samples, bovine serum albumin (BSA) was included at a final concentration of 1% to minimize nonspecific binding. Samples were rotated for 1 h at 4°C, followed by washing twice with 1 mL ice-cold PBS and centrifuged at 500 × *g* for 5 min between washes. The resulting pellets were lysed by adding 4× Laemmli sample buffer and boiled for 5 min at 95°C. An equal concentration of each lysate was resolved on a 12.5% SDS-PAGE gel, followed by transfer to a PVDF membrane. Western blot was performed using primary anti-PsaA serum (1:5,000), which detects both PsaA and PsaA-derived peptides, followed by HRP-conjugated goat anti-mouse IgG secondary antibody (1:10,000). Protein detection was performed using the SuperSignal West Pico PLUS Chemiluminescent Substrate.

### Quantitative measurement with flow cytometry

For flow cytometry, cell-protein/peptide binding was performed using the protocol stated above. After the binding assay, cells were incubated with anti-PsaA serum as the primary antibody in eBioscience Flow Cytometry Staining Buffer (ThermoFisher Scientific, Cat# 00-4222-26) at a 1:100 dilution for 30 min at 4°C in the dark. Next, the cells were washed twice in 1 mL ice-cold PBS and centrifuged at 500 × *g* for 5 min between washes. Cells were then stained with secondary antibody goat anti-mouse IgG (H+L), human ads-Alexa Fluor 488 (SouthernBiotech, Cat#1040-30) at 1:200 dilution for 30 min at 4°C in the dark. Following the staining, cells were washed twice in 1 mL ice-cold PBS and centrifuged at 500 × *g* for 5 min between washes. Cells were resuspended in 1 mL ice-cold PBS, and flow cytometry was performed in Attune Acoustic Focusing Cytometer (Applied Biosystems). The experiment was repeated at least three times for data acquisition.

### Adhesion assay

Adhesion assays were performed using the human A549 epithelial cell line (ATCC CCL-185). Cells were grown in F12K medium (ATCC 30-2004) supplemented with 10% FBS and penicillin/streptomycin (Cytiva, Cat# SV30010) until 80%–90% confluency. Prior to adhesion assays, cells were washed 2× with PBS and then treated with Gibco Cell Dissociation Buffer, enzyme-free, PBS (ThermoFisher Scientific, Cat# 13151014) and diluted to a final concentration of 1 × 10^5^ cells/ml. Unencapsulated *S. pneumoniae* TIGR4 (JS1 strain) bacteria were applied to the cells at a concentration of 5 × 10^5^ cells/ml (5:1 ratio of bacteria per cell). Anti-S1, anti-S3, and non-immune mouse serum were pre-incubated with the bacteria on ice for 30 min. After pre-incubation, each serum and bacteria mixture was added to A549 cells and incubated at 37°C for 30 minutes with rotation. After 30 min, cells were washed three times with D-PBS with calcium and magnesium (D-PBS +Ca/Mg), trypsinized, and suspended in Triton X-100 (0.025% final concentration). Samples were diluted 1:10 in PBS and plated on TSA + 5% defibrinated sheep blood agar. Plates were incubated at 37°C with 5% CO_2_ overnight. Each experimental condition was plated in triplicate for quantification and repeated at least five times independently.

### Statistical analysis

Quantitative data are presented as mean ± standard deviation (SD). Statistical analysis was performed using GraphPad Prism. Western blot densitometry values, normalized to the internal loading control from four independent experiments shown in Fig. 3c, were analyzed by one-way analysis of variance (ANOVA), followed by Dunnett’s multiple-comparisons test. As shown in Fig. 5, bacterial adherence when blocked with anti-S1 or anti-S3 sera was normalized to control mouse serum, and one-way ANOVA followed by Tukey’s multiple-comparison test was performed from five independent experiments. *P*-value <0.05 was considered statistically significant.

## Data Availability

Any data or materials generated in this manuscript are available upon request, in compliance with the ASM Data Policy.

## SUPPLEMENTAL MATERIAL

Supplemental Material msphere.00232-26-s0001.docx Supplemental figures and table.

ASM does not own the copyrights to Supplemental Material that may be linked to, or accessed through, an article. The authors have granted ASM a non-exclusive, world-wide license to publish the Supplemental Material files. Please contact the corresponding author directly for reuse.
