# Supplementary material for: Identification of the Annexin A2-interacting domain of pneumococcal PsaA
Source: mSphere. 2026 Apr 30;11(5):e00232-26. doi: 10.1128/msphere.00232-26 (PMC13203966; doi:10.1128/msphere.00232-26)
Supplement: Supplemental Material — Supplemental figures and table. [file msphere.00232-26-s0001.docx]

**Supplemental Figures:**

**
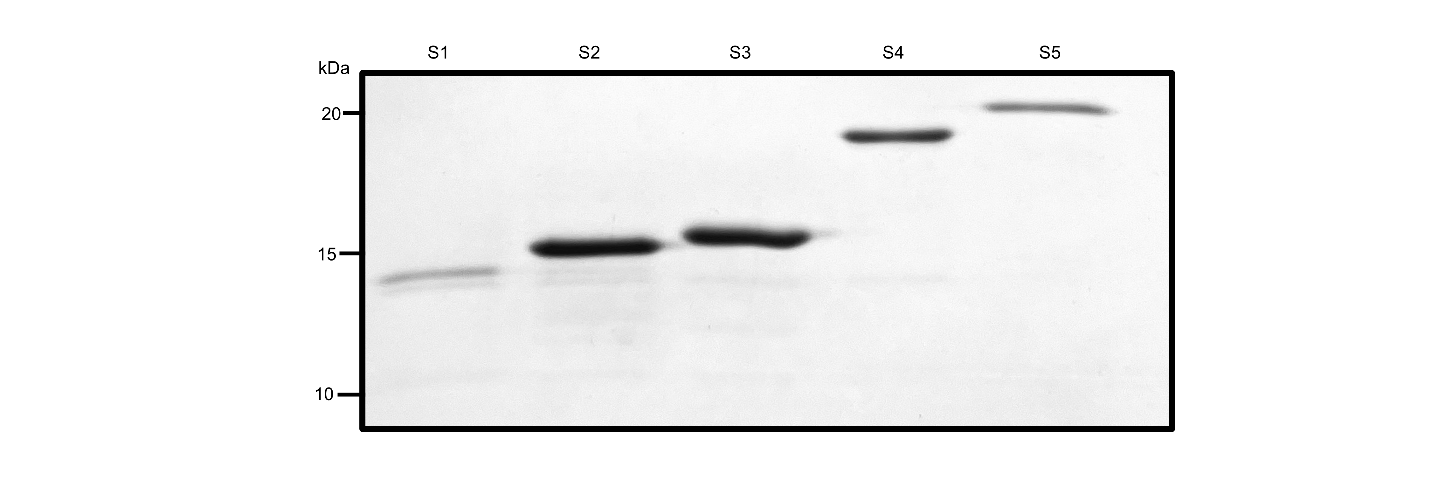
**

**Figure S1: Coomassie staining of peptides on SDS-PAGE gel purified by affinity purification, cloned into the pET100 vector**

PsaA-derived peptides at their expected molecular weight S1 (≈12.79 kDa), S2 (≈14.55 kDa), S3 (≈17.62 kDa), S4 (≈19.21 kDa), S5 (≈21.58 kDa)

**
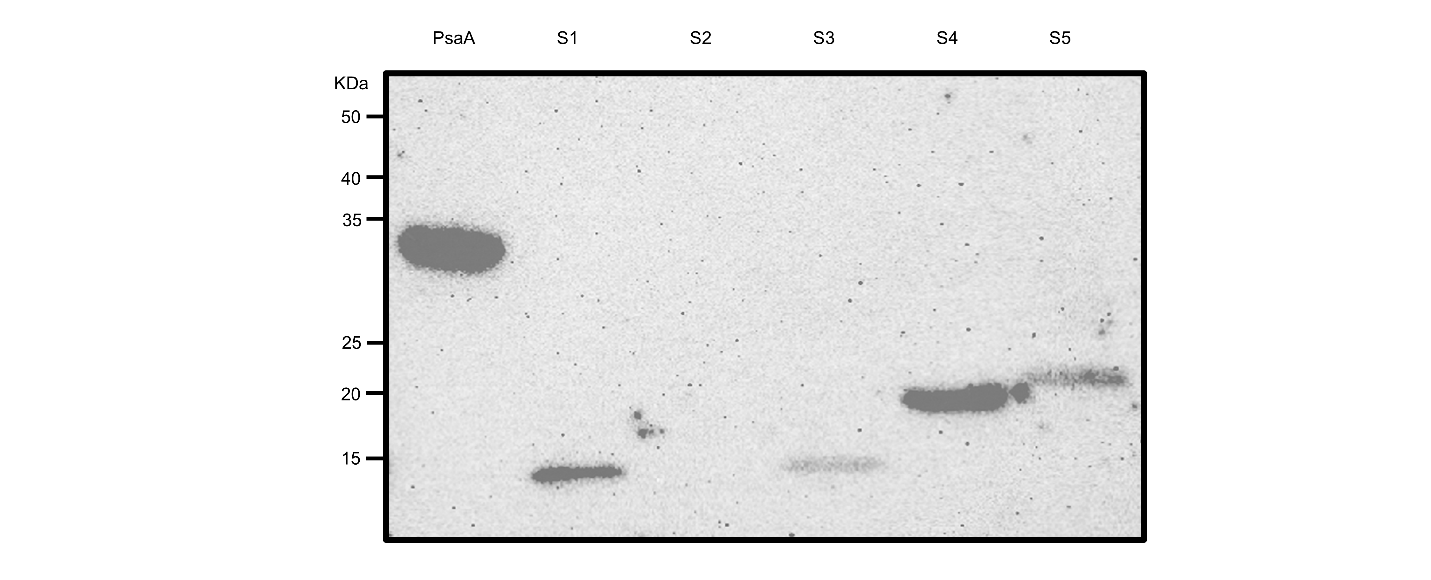
**

**Figure S2: Detection of full-length PsaA and PsaA-derived peptides using anti-PsaA serum**

Full-length PsaA and peptides S1, S3-S5 were detected with 1:2500 anti-PsaA serum and 1:10000 goat-anti-mouse HRP-conjugated secondary antibody in a western blot


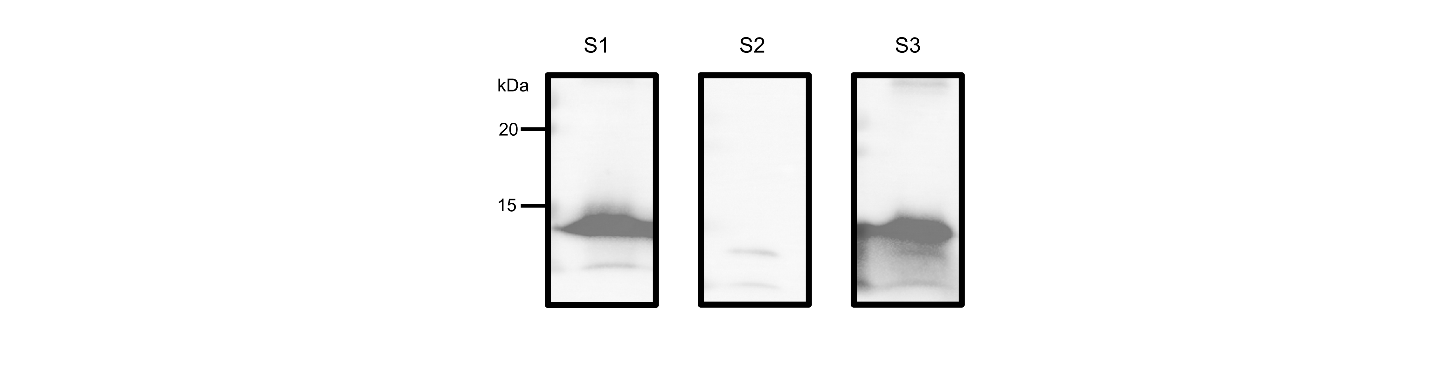


**Figure S3: Normalization of Anti-Peptide Serum Titer**

Anti-peptide serum titer was normalized by loading an equal concentration of peptides on a SDS-PAGE gel and performing a western blot. S1 and S3 peptides could be detected with 1:2500 dilution of serum. S2 peptide could not be detected due to its non-immunogenic nature.

**Supplemental Table 1: PsaA Peptides Primers**

| Peptide | Forward Primer | Reverse Primer |
| --- | --- | --- |
| S1 | 5′-CACCGCTAGCGGAAAAAAAGATACAACT-3′ | 5′-TTAAAACCAAGCATTGCCACCTG-3′ |
| S2 | 5′-CACCACAAAATTGGTAGAAAATGCCAAGAAAACT-3′ | 5′-TTATTTATCCTTACTTTCTTTATCAAGTTTGTCTAACTT-3′ |
| S3 | 5′-CACCTTTAATAAGATCCCTGCTGAAAAGAAACTC-3′ | 5′-TTATTTTGCCAATCCTTCAGCAATCTT-3′ |
| S4 | 5′-CACCGCTAGCGGAAAAAAAGATACAACT-3′ | 5′-TTATTGTTTGGCGATATTTTTAGCAAAAATAATACC-3′ |
| S5 | 5′-CACCTTGAGCGCCAAAGACCCTAAC-3′ | 5′-TTATTTTGCCAATCCTTCAGCAATCTT-3′ |
